# 6-Hydroxy-3-Succinoylpyridine Hydroxylase Catalyzes a Central Step of Nicotine Degradation in *Agrobacterium tumefaciens* S33

**DOI:** 10.1371/journal.pone.0103324

**Published:** 2014-07-23

**Authors:** Huili Li, Kebo Xie, Haiyan Huang, Shuning Wang

**Affiliations:** 1 State Key Laboratory of Microbial Technology, Shandong University, Jinan, PR China; 2 Institute of Basic Medicine, Shandong Academy of Medical Science, Jinan, PR China; National Research Council of Italy, Italy

## Abstract

Nicotine is a main alkaloid in tobacco and is also the primary toxic compound in tobacco wastes. It can be degraded by bacteria via either pyridine pathway or pyrrolidine pathway. Previously, a fused pathway of the pyridine pathway and the pyrrolidine pathway was proposed for nicotine degradation by *Agrobacterium tumefaciens* S33, in which 6-hydroxy-3-succinoylpyridine (HSP) is a key intermediate connecting the two pathways. We report here the purification and properties of an NADH-dependent HSP hydroxylase from *A. tumefaciens* S33. The 90-kDa homodimeric flavoprotein catalyzed the oxidative decarboxylation of HSP to 2,5-dihydroxypyridine (2,5-DHP) in the presence of NADH and FAD at pH 8.0 at a specific rate of about 18.8±1.85 µmol min^−1 ^mg protein^−1^. Its gene was identified by searching the N-terminal amino acid residues of the purified protein against the genome draft of the bacterium. It encodes a protein composed of 391 amino acids with 62% identity to HSP hydroxylase (HspB) from *Pseudomonas putida* S16, which degrades nicotine via the pyrrolidine pathway. Considering the application potential of 2,5-DHP in agriculture and medicine, we developed a route to transform HSP into 2,5-DHP with recombinant HSP hydroxylase and an NADH-regenerating system (formate, NAD^+^ and formate dehydrogenase), via which around 0.53±0.03 mM 2,5-DHP was produced from 0.76±0.01 mM HSP with a molar conversion as 69.7%. This study presents the biochemical properties of the key enzyme HSP hydroxylase which is involved in the fused nicotine degradation pathway of the pyridine and pyrrolidine pathways and a new green route to biochemically synthesize functionalized 2,5-DHP.

## Introduction

Microbial degradation of nicotine has drawn an increasing interest recently since it has various biochemical and physiological mechanisms and represents a promising biological method to treat the tobacco leaves and wastes [1]–[6]. Nicotine, the most abundant alkaloid in tobacco plants, makes people addicted to tobacco and leads to a number of diseases such as cancer and pulmonary disease [7], [8]. It is also the major toxic compound in tobacco wastes, which are largely produced during tobacco manufacturing process and all activities of tobacco use, and could cause serious environmental problems [9], [10]. In addition, as one of the important traditional cash crops, tobacco is planted in a big scale in many countries. However, WHO Framework Convention on Tobacco Control has been adopted by most countries in the world, because of which some new technologies to utilize tobacco are necessary to be developed in the near future. Considering the high content of nicotine in tobacco leaves and wastes ranging from 2% to 8% [9], [11], chemists have already tried to use it as a starting material to produce some chemicals of pharmaceutical importance [12]–[14]. Nicotine can also be modified into important functionalized pyridines by biocatalytic processes that are difficult to synthesize via chemical methods [15]. For example, it can be transformed by *Arthrobacter* sp. and *Pseudomona*s sp. into valuable chemicals such as 6-hydroxy-nicotine, 6-hydroxy-3-succinoylpyridine (HSP) and 2,5-dihydroxypyridne (2,5-DHP), which are important precursors for synthesis of drugs and insecticides [16]–[18].

A number of microorganisms able to degrade nicotine have been isolated and characterized till now, including bacteria, actinomycetes and fungi [1], [2], [19], [20], among which only a few were studied for their biochemical pathways to degrade nicotine. The proposed pathways so far can be summarized mainly into three groups: the demethylation pathway used by fungi such as *Aspergillus oryzae*; the pyridine pathway used by Gram-positive bacterium *Arthrobacter* sp.; and the pyrrolidine pathway used by Gram-negative bacterium *Pseudomonas* sp. [6], [21], [22]. The biochemical and molecular mechanism involved in the pyridine and pyrrolidine pathways have been already well elucidated or are being studied [1], [3], [6], [21], [23]. For other microorganisms, however, the biochemical pathway and molecular mechanism in nicotine catabolism are seldom reported.

**Figure 1:**
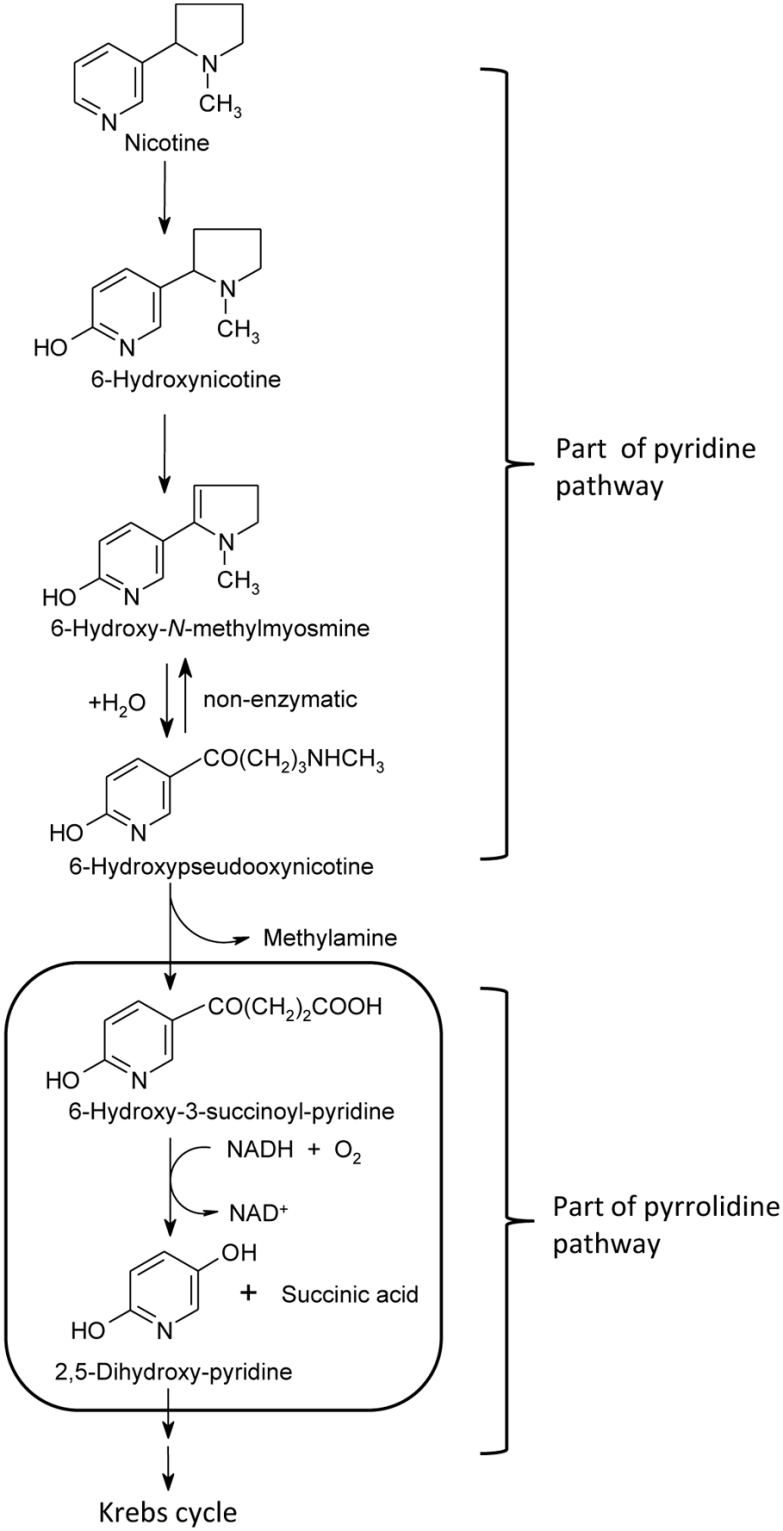
Proposed pathway for nicotine degradation by *A. tumefaciens* S33. The steps from nicotine to 6-hydroxy-pseudooxynicotine are same to part of the pyridine pathway, and the step catalyzed by 6-hydroxy-3-succinoylpyridine hydroxylase, which is indicated in the box, is same to the pyrrolidine pathway.

Previously, a novel pathway different from the reported pathways mentioned above in *Agrobacterium tumefaciens* S33 was characterized and proposed, that is, by way of the identification of its intermediates and measurement of key enzymes activities in cell extracts and partially enriched enzymes [20], [24]. In this novel pathway (Figure 1), nicotine is firstly transformed into 6-hydroxy-pseudooxynicotine via the pyridine pathway through 6-hydroxy-L-nicotine and 6-hydroxy-*N*-methylmyosmine, and then it turns to the pyrrolidine pathway with the formation of HSP and 2,5-DHP. HSP hydroxylase, one of the key enzymes, was partially enriched to 7 folds from *A. tumefaciens* S33, which has also been found as the key enzyme in the pyrrolidine pathway in *Pseudomonas putida* S16 [21], but its biochemical properties and encoding gene in *A. tumefaciens* S33 was still unknown.

In this study, the HSP hydroxylase was purified from *A. tumefaciens* S33, its biochemical properties was characterized, and its encoding gene was identified by determination of the N-terminal amino acids sequence and genome survey. Because 2,5-DHP, the product of the reaction catalyzed by HSP hydroxylase, is a valuable precursor for the chemical synthesis of 5-aminolevulinic acid, which is applied as a plant growth hormone, a herbicide and in cancer therapy, an efficient process to transform HSP into 2,5-DHP was developed here with heterologously expressed HSP hydroxylase and NADH-regenerating system (formate, NAD^+^ and formate dehydrogenase from *Candida boidinii*).

## Materials and Methods

### Bacterial strain and growth

*Agrobacterium tumefaciens* S33, isolated from tobacco plant rhizosphere and deposited at China Center for Type Culture Collection (CCTCC) under accession number CCTCC M 206131 [20], was grown in nicotine medium [24] plus 1.0 g l^−1^ glucose, 0.2 g l^−1^ (NH_4_)_2_SO_4_ and 1.0 g l^−1^ yeast extract at 30°C. Tungstate was omitted from the trace elements solution because it is the specific antagonist of molybdate and would inhibit the molybdenum-containing nicotine dehydrogenase [25]. Nicotine (≥99% purity, Fluka) was added into the media with the final concentration of 1.0 g l^−1^ prior to filter sterilization before inoculation. The pre-culture was obtained by growing the bacteria in nicotine medium with nicotine as the sole source of carbon and nitrogen. Cells were harvested in the late of exponential phase.

### Purification of 6-hydroxy-3-succinoylpyridine hydroxylase (HSP hydroxylase)

Ten grams of wet cells obtained from 3 liters of culture were re-suspended in 50-ml 50 mM Tris-HCl (pH 8.0) and disrupted by an ultrasonic liquid processor (Vibra-Cell VCX 500, Sonics and Materials, amplitude 30%, 20 min, pulse on 6 s and pulse off 6 s) in an ice/water bath. Unbroken cells and cell debris were removed by centrifugation at 30,000×*g* and 4°C for 30 min. The supernatant was used as cell extract for enzyme purification.

All chromatography steps were carried out using an ÄKTA Basic10 chromatography system (GE-Healthcare). All buffers contained 5 µM FAD, which could slow down the activity loss during purification. Firstly, the cell extract was subjected to ammonium sulfate precipitation by slowly adding saturated ammonium sulfate solution to different final concentrations at 4°C. After gently stirred for 30 min, the precipitate was recovered by centrifugation at 30,000×*g* and 4°C for 20 min. Most HSP hydroxylase activity was found in the fraction obtained by precipitating between 40% and 60% saturation of ammonium sulfate, which was collected by centrifugation. The precipitate was dissolved in 5 ml 50 mM Tris-HCl (pH 8.0) containing 1.0 M ammonium sulfate. After removing the undissolved proteins by centrifugation, the supernatant was applied on a Butyl Sepharose 6 Fast Flow (high sub, 16 mm×10 cm, 20 ml) equilibrated with 50 mM Tris-HCl (pH 8.0) containing 1.0 M ammonium sulfate. The column was eluted at a 4 ml min^−1^ flow rate with 5 column volumes of each 1.0, 0.8, 0.6, 0.4, 0.2 and 0 M ammonium sulfate step gradients in the same buffer. HSP hydroxylase was eluted at an ammonium sulfate concentration of 0.8 M. The fractions containing the activity were pooled, concentrated, and desalted by the use of an Amicon filter (30-kDa-cutoff). Then the sample was applied on a DEAE Sepharose Fast Flow (16 mm×10 cm, 20 ml) equilibrated with 50 mM Tris-HCl (pH 8.0). The column was eluted at a 4 ml min^−1^ flow rate with 5 column volumes of each 0.1, 0.2, 0.3, 0.4, and 0.5 M NaCl step gradients in the same buffer. HSP hydroxylase was eluted at a NaCl concentration of 0.2 M. The fractions containing the activity were pooled and desalted, and concentrated. Then the concentrate was applied to a Source 30 Q column (16 mm×10 cm, 20 ml) equilibrated with 50 mM Tris-HCl (pH 8.0). The column was eluted at a 3 ml min^−1^ flow rate with the same procedure as DEAE Sepharose Fast Flow step. HSP hydroxylase was eluted at a NaCl concentration of around 0.2 M. After pooling and concentrating the fractions with the activity, the sample was loaded on a 24-ml Superdex 200 column, which was equilibrated with 50 mM Tris-HCl (pH 8.0) containing 150 mM NaCl prior to loading the sample and eluted with the same buffer. The fraction size collected was set at 1 ml per tube. HSP hydroxylase activity was found in a single peak at 14 ml fraction tube. Fractions with the activity collected during the purification were analyzed by sodium dodecyl sulfate-polyacrylamide gel electrophoresis (SDS-PAGE) using a 12.5% gel according to the standard procedure in a Bio-Rad Mini-Protean III Cell (Laemmli 1970) and stained with Coomassie brilliant blue. The protein concentration was measured by the Bradford method with bovine serum albumin as the standard.

### Enzyme assay

The activity of HSP hydroxylase was measured based on the finding by Wang et al. [22], [24] and Tang et al. [21], [23] that HSP hydroxylase is a NADH-dependent enzyme. The assay was performed at 30°C in a quartz cuvette (1 cm light path) filled with 1-ml reaction mixture. The assay mixture contained 12 µM FAD, 1 mM HSP, 0.5 mM NADH and 50 mM Tris-HCl (pH 8.0). HSP was prepared from the broth of nicotine degrading *P. putida* S16 as described previously [18] with 98% purity verified by HPLC analysis. The reaction was initiated by adding enzyme, and NADH oxidation was spectrophotometrically monitored using an Ultrospec 2100 pro UV/Visible Spectrophotometer (GE Healthcare, USA) by the decrease in absorption at 380 nm (ε = 1.2 mM^−1 ^cm^−1^). The wavelength of 380 nm instead of 340 nm was chosen because neither HSP nor the product 2,5-DHP has absorption at 380 nm. One unit was defined as the oxidation of 1 µmol NADH per min.

The activities of *p*-nitrophenol monooxygenase and 6-hydroxynicotinic acid 3-monooxygenase were measured according to the previous description [16], [26].

### N-terminal amino acid sequence determination of HSP hydroxylase

After analyzed by SDS-PAGE according to the standard procedure, the target protein was transferred to PVDF membrane by electroblotting and visualized with Coomassie brilliant blue solution. Then the corresponding bands were excised and the N-terminal amino acid sequence of the protein was determined by automated Edman degradation using an Applied Biosystems 477A protein/peptide sequencer and the protocol given by the manufacturer.

### Genome survey of *A. tumefaciens* S33

*A. tumefaciens* S33 genomic DNA was extracted and purified with Wizard Genomic DNA Purification Kit (Promega). The genome sequence of *A. tumefaciens* S33 was determined by BGI (Shenzhen, China) using the Illumina GA system with a paired-end library. The short reads were assembled with SOAPdenovo, version 1.05 [27]. Gene prediction and annotation were carried out using the RAST annotation server [28].

### Isolation of total RNA and RT-PCR

*A. tumefaciens* S33 was grown in nicotine medium [20] containing 1.0 g l^−1^ nicotine as the sole source of carbon and nitrogen, or in a medium containing 1.0 g l^−1^ glucose, 0.2 g l^−1^ (NH_4_)_2_SO_4_ and 1.0 g l^−1^ yeast extract at 30°C. The cells were harvested at the early-exponential phase when the optical density at 620 nm reached around 0.4 in nicotine medium or 0.7 in glucose and ammonium medium. Total RNA was isolated from cell pellets using RNAprep pure Cell/Bacteria Kit (TIANGEN Biotech, China) according to the manufacturer’s protocol. Contaminating DNA was removed by treatment with DNase I (RNase-free) provided in the kit at 37°C for 30 min. Total cDNA was synthesized with TransScript First-Strand cDNA Synthesis SuperMix (Beijing TransGen Biotech, China) using 0.7 µg of RNA and random primers provided in the kit according to the manufacturer’s manual. The HSP hydroxylase gene was amplified in 20 µl PCR mixture with 95 ng of total cDNA as the template. The amplification condition used for PCR reaction was as follows: 94°C for 5 min; 33 cycles of 94°C for 30 s, 60°C for 30 s and 72°C for 1 min; and then 72°C for 10 min. A full length of HSP hydroxylase gene (1,176 bp) was obtained by using the same primers for heterologous expression (see below). The amplification with genomic DNA and total RNA as the template and the same PCR reaction procedure were used for positive control and negative control, respectively.

### Heterologous expression and purification of His-tagged HSP hydroxylase

The HSP hydroxylase gene was amplified by PCR with high-fidelity FastPfu DNA polymerase (Shanghai ShineGene Molecular Bio-tech, China) by the use of *A. tumefaciens* S33 genomic DNA as a template. The following primers were used: 5′- CGGGATCCTATGAGCGCACATGTCGTTGTCGT (forward primer; the BamHI restriction site is underlined) and 5′-TATCTCGAGTCAATATACTGTCCGCATCTGTTCGTGG (reverse primer; the XhoI restriction site is underlined). The blunt PCR product was ligated into pEASY-Blunt cloning vector (Beijing TransGen Biotech, China), which was subsequently transformed into *E. coli* Mach 1-T1 cells (Invitrogen). After amplification, the construct was digested by BamHI and XhoI, and the target fragment was ligated into expression vector pETDuet-1, which had been digested by the same restriction endonucleases. The new construct pETDuet-hsph was introduced into Mach 1-T1 cells again and verified by DNA sequencing. It was then transformed into *E. coli* BL21(DE3) for expression. The cells were grown in 1 liter of LB medium containing 50 mg of ampicillin liter^−1^ at 16°C at a low stirring speed (100 rpm). IPTG was added to a final concentration of 0.1 mM immediately after inoculation (OD_620nm_, between 0.2 and 0.3). After incubation for 24 h, the cells were harvested by centrifugation. The induced cells were washed twice with 50 mM Tris-HCl buffer (pH 8.0) and stored at −20°C until use. Via this method, around 60% recombinant protein product were found to be soluble in the supernatant, while most products were in the inclusion bodies when it was induced in classic method with 0.5 mM IPTG at 37°C.

For purification of the His-tagged HSP hydroxylase, the *E. coli* cells (6 g, wet weight) were re-suspended in 30 ml 50 mM Tris-HCl buffer (pH 8.0), and disrupted by sonification in an ice/water bath. Cell debris was removed by centrifugation at 30,000×*g* and 4°C for 30 min. The clarified supernatant was applied on a 5-ml His Trap HP column (GE Healthcare). The recombinant protein was eluted with 50 mM Tris-HCl buffer (pH 8.0) containing imidazole in a linear gradient ranging from 10 mM to 100 mM in a total gradient volume of 100 ml at a flow rate of 4 ml min^−1^. The fractions with the activity were pooled and applied on a Source 30Q column (16 mm×10 cm, 20 ml). The column was eluted with 50 mM Tris-HCl buffer (pH 8.0) containing 0.5 M NaCl in a linear gradient for 20 column volumes at a flow rate of 4 ml min^−1^. The HSP hydroxylase activity was recovered in the fractions eluted with 0.15 M NaCl. The protein was concentrated by ultrafiltration with an Amicon filter (30-kDa-cutoff) and stored at −20°C. All the buffers used were supplemented with 5 µM FAD in order to avoid its disassociation from the enzyme during purification.

### Heterologous expression of formate dehydrogenase (Fdh) in *E. coli* and purification of His-tagged Fdh

The recombinant pETDuet-1 harboring *fdh* from *Candida boidinii* was a gift of Professor Cuiqing Ma from Shandong University. The *fdh* gene was cleaved with NdeI and XhoI and ligated into pACYCDuet-1. And the new construct pACYCDuet-fdh was then transformed into *E. coli* BL21(DE3) for expression. Induction and purification of His-tagged Fdh was performed with the same procedure as His-tagged HSP hydroxylase. The activity was measured according to what had been previously described with modification [29]. Briefly, the 0.8 ml-mixture contained 1.67 M sodium formate, 1.67 mM NAD^+^ and 50 mM sodium phosphate buffer (pH 8.0). The reaction was carried out at 25°C, started by adding formate dehydrogenase, and monitored by determining NAD^+^ reduction at 340 nm (ε = 6.2 mM^−1 ^cm^−1^). One unit (U) was defined as the reduction 1 µmol NAD^+^ per min.

### Transformation of HSP into 2,5-DHP

Two methods were tried to convert HSP to 2,5-DHP. (i) Transformation of HSP into 2,5-DHP with whole cells of *E. coli* BL21(DE3) harboring pETDuet-hsph and pACYCDuet-fdh. Firstly the constructs pETDuet-hsph and pACYCDuet-fdh were co-transformed into the competent *E. coli* BL21(DE3) cells. The recombinant *E. coli* BL21(DE3) was then grown and induced with the same procedure as expressing His-tagged HSP hydroxylase. After incubation for 20 h, the cells were harvested by centrifugation and washed twice with 50 mM Tris-HCl buffer (pH 8.0), then were used as catalysts to perform the transformation reaction. The reaction mixture contained 50 mM sodium phosphate buffer (pH 8.0), 0.75 mM HSP, 1.67 mM NAD^+^, 83.5 mM sodium formate and 1.5 g liter^−1^ wet cells of recombinant *E. coli* BL21(DE3) prepared as described above. The reaction was carried out at 25°C with gentle shaking at 60 rpm. Samples were withdrawn at regular intervals and analyzed by TLC and HPLC after those cells were removed by centrifugation. (ii) Enzymatic transformation of HSP into 2,5-DHP with recombinant HSP hydroxylase. The reaction was performed in a 10-ml tube containing 5 ml of mixture composed of 50 mM sodium phosphate buffer (pH 8.0), 0.75 mM HSP, NADH-regenerating system (1.67 mM NAD^+^, 83.5 mM sodium formate, 5 units of formate dehydrogenase), and 8 units of recombinant HSP hydroxylase. The reaction mixture was sampled at regular intervals. The reaction was monitored by TLC, and the amount of substrates and products was determined by HPLC according to previous description [24] with small modification of a mixture of methanol and 1 mM formic acid (87∶13, v/v) used as the mobile phase and the flow rate at 0.5 ml min^−1^.

### Analytical methods

The reaction mixture of enzyme assay was added equal volume of ethanol and shook for 10 min. After centrifugation at 30,000×*g* and 4°C for 20 min, the supernatant was used for identification of products with liquid chromatography-mass spectrometry (LC-MS) analysis. LC-ESI-MS data were obtained using a Finnigan Surveyor MSQ single quadrupole electrospray ionization mass spectrometer coupled with a Finnigan Surveyor HPLC (Finnigan/Thermo Electron Corporation, San Jose, CA, USA). Negatively charged ions were detected. The HPLC system was performed with a 20RBAX Eclips XDB-C18 column (column size, 150×4.6 mm; partical size, 5 µm; Agilent, USA) and a photodiode array (PDA) detector. A mixture of methanol and 1 mM formic acid (85∶15, v/v) was used as the mobile phase and the flow rate was set at 0.5 ml min^−1^. The sample was also analyzed by GC-MS after lyophilization and silylation with *N,O*-Bis(trimethylsilyl) trifluoroacetamide (BSTFA, Supelco) as described [24].

### Nucleotide sequence accession number

The nucleotide sequence of HSP hydroxylase gene reported in this study has been deposited in the GenBank database under the accession number KJ129609.

## Results and Discussion

### Purification and characterization of HSP hydroxylase from *A. tumefaciens* S33

It was previously found that cell extracts of *A. tumefaciens* S33 grown on nicotine as the sole source of carbon and nitrogen catalyzed NADH-dependent hydroxylation of HSP with a specific activity of 0.15 U/mg to 0.19 U/mg [24]. The activity was enriched for 7 folds after ammonia sulfate fractionation and DEAE Sepharose treatment. In this study, the bacterium was grown in nicotine medium supplemented with glucose and yeast extract in order to obtain more cells. Based on the previous study [20], the cells were harvested in the late exponential phase to ensure that the enzymes involved in nicotine degradation were induced. Cell extracts prepared in this way presented a NADH-dependent HSP hydroxylase activity of 0.16 U/mg and a relatively low NADPH-dependent HSP hydroxylase activity of 0.04 U/mg. Then HSP hydroxylase was purified from such cell extracts (Table 1).

**Table 1:** Purification of HSP hydroxylase from *A. tumefaciens* S33.

Step	Total protein (mg)	Total activity (unit)	Specific activity(unit/mg)	Yield (%)	Fold
Cell extract	454.6	73.8	0.16	100	1
40–60% (NH4)2SO4 precipitation	303.8	54.7	0.18	74	1.1
Butyl Sepharose	15.2	30.5	2.0	41.3	12.5
DEAE Sepharose	6.4	26.4	4.1	35.8	25.6
Source 30Q	2.6	24.8	9.4	33.6	58.7
Superdex 200	1.9	19.6	10.3	26.6	64.4

Purification was achieved by ammonia sulfate fractionation, hydrophobic chromatography (Butyl Sepharose), anion-exchange chromatography (DEAE Sepharose and Source 30Q) and gel filtration (Superdex 200). During the purification, the activities were determined by spectrophotometrically monitoring HSP-dependent NADH oxidation at 380 nm. The enzyme was purified 64-fold, with a yield of 27% and a NADH-dependent specific activity of 10.3 U/mg (Table 1).

During purification, all the buffers were supplemented with 5 µM FAD. When FAD was omitted, the purification yield was very low. FAD could not be substituted by FMN. Because FAD was added to all the buffers during purification, the amount of it in the purified enzyme was not determined.

**Figure 2:**
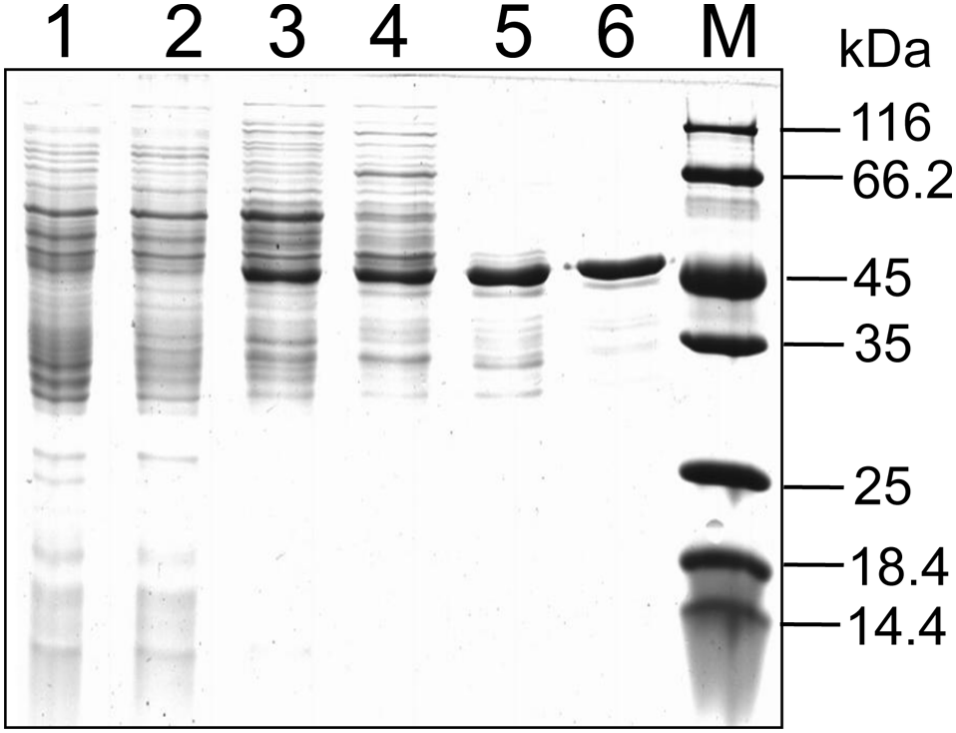
Purification of HSP hydroxylase from *A. tumefaciens* S33. Lane 1, cell extract; lane 2, ammonia sulfate precipitation; lane 3, Butyl Sepharose; lane 4, DEAE Sepharose; lane 5, Source 30Q; lane 6, Superdex 200; and M, protein marker.

SDS-PAGE of the purified enzyme showed one protein with a molecular mass of around 45 kDa (Figure 2). The apparent molecular mass of the enzyme determined by gel exclusion chromatography was near 90 kDa, and this indicates that the native enzyme is a homodimer.

### Identification of HSP hydroxylase gene in the genome of *A. tumefaciens* S33

The N-terminal amino acid sequence of the purified HSP hydroxylase was determined by automated Edman degradation to be SAHVVVVGGGPTGLLTTLGL. Its encoding gene was identified by searching the N-terminal amino acid sequence against the genome draft sequence of *A. tumefaciens* S33, which was automatically annotated on RAST online server. HSP hydroxylase gene (1,176 bp) was found to encode a 44-kDa protein composed of 391 amino acids, which has 62% identity to HspB [21] and no significant similarity with HspA [23]. HspA and HspB are two HSP hydroxylase isozymes involved in nicotine degradation in *P. putida* S16. In addition, HSP hydroxylase from *A. tumefaciens* S33 also shows high identity in protein sequence to *p*-nitrophenol monooxygenase from *Pseudomonas* sp. WBC-3 (38%) [26], *Pseudomonas* sp. NyZ402 (37%) [30] and *P. putida* DLL-E4 (37%) [31]. The three *p*-nitrophenol monooxygenases are almost identical in protein sequence (more than 85% identity) and convert *p*-nitrophenol to *p*-benzoquinone in the presence of NAD(P)H and FAD in *p*-nitrophenol degradation. And HSP hydroxylase has relatively low identity to 6-hydroxynicotinic acid 3-monooxygenase from *P. putida* KT2440 (17%) [32] and *P. fluorescens* TN5 (16%) [16], which catalyzes oxidative decarboxylation of 6-hydroxynicotinic acid to render 2,5-DHP in nicotinic acid degradation. Conserved domains analysis in NCBI shows that N-terminal domain of HSP hydroxylase from *A. tumefaciens* S33 (amino acids 22–169) belongs to PRK07608 superfamily (ubiquinone biosynthesis hydroxylase family protein), where a GADGA motif was found (amino acids 156–160), and HSP hydroxylase contains a FAD-binding domain (pfam01494, amino acids 22–333). The feature is also found in HspB from *P. putida* S16 [21]. Alignment of HSP hydroxylase from *A. tumefaciens* S33 with the proteins mentioned above and other FAD-dependent monooxygenases indicates that HSP hydroxylase contains the conserved motifs for FAD and NAD(P)H binding, including GxGxxG, DGxcSxhR, and GxhhLhGDAAHxxxPxxGxGxNxxxxDxxxL (x = all residues; c = charged residues; h = hydrophobic residues), that are associated with the hydroxylase activity [33]. However, HSP hydroxylase from *A. tumefaciens* S33 did not show any detectable activity with *p*-nitrophenol or 6-hydroxynicotinate as substrate in the presence of NAD(P)H and FAD.

**Figure 3:**
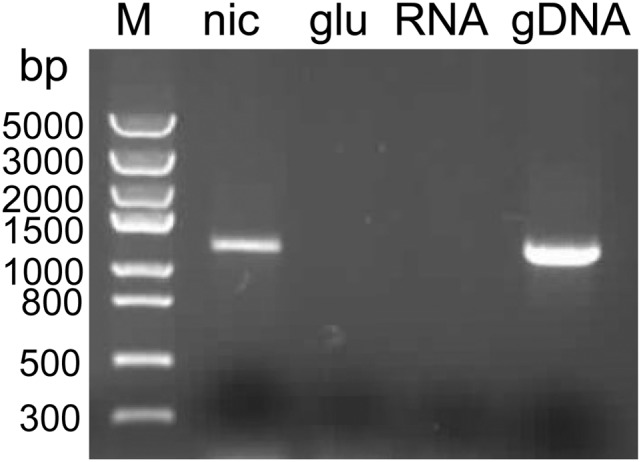
RT-PCR analysis of HSP hydroxylase gene transcription in *A. tumefaciens* S33. The cells were grown in the medium with nicotine as the sole source of carbon and nitrogen (lane nic) and in the glucose and ammonium medium (lane glu), respectively. The gene is 1,176 bp long. Lane RNA, negative control, the total RNA isolated from the culture grown in the medium with nicotine as the sole source of carbon and nitrogen as the template; lane gDNA, positive control, the genomic DNA isolated from the culture grown in the glucose and ammonium medium as the template.

Moreover, in order to confirm whether the gene is really involved in the nicotine degradation in *A. tumefaciens* S33, RT-PCR analysis was performed. Total RNA was isolated from cultures grown in the medium with nicotine as the sole source of carbon and nitrogen and in the medium with glucose and ammonium sulfate as the sources of carbon and nitrogen. After reverse transcription with random primers, the full length of HSP hydroxylase gene (1,176 bp) was amplified with specific primers. As shown in Figure 3, RT-PCR resulted in the specific full fragment of HSP hydroxylase gene when the strain was grown in the medium with nicotine as the sole source of carbon and nitrogen. In contrast, PCR product was not detected when the strain was grown in the glucose and ammonium medium, and this indicates that expression of HSP hydroxylase gene in *A. tumefaciens* S33 is induced in the presence of nicotine.

### Catalytic properties of HSP hydroxylase

**Figure 4:**
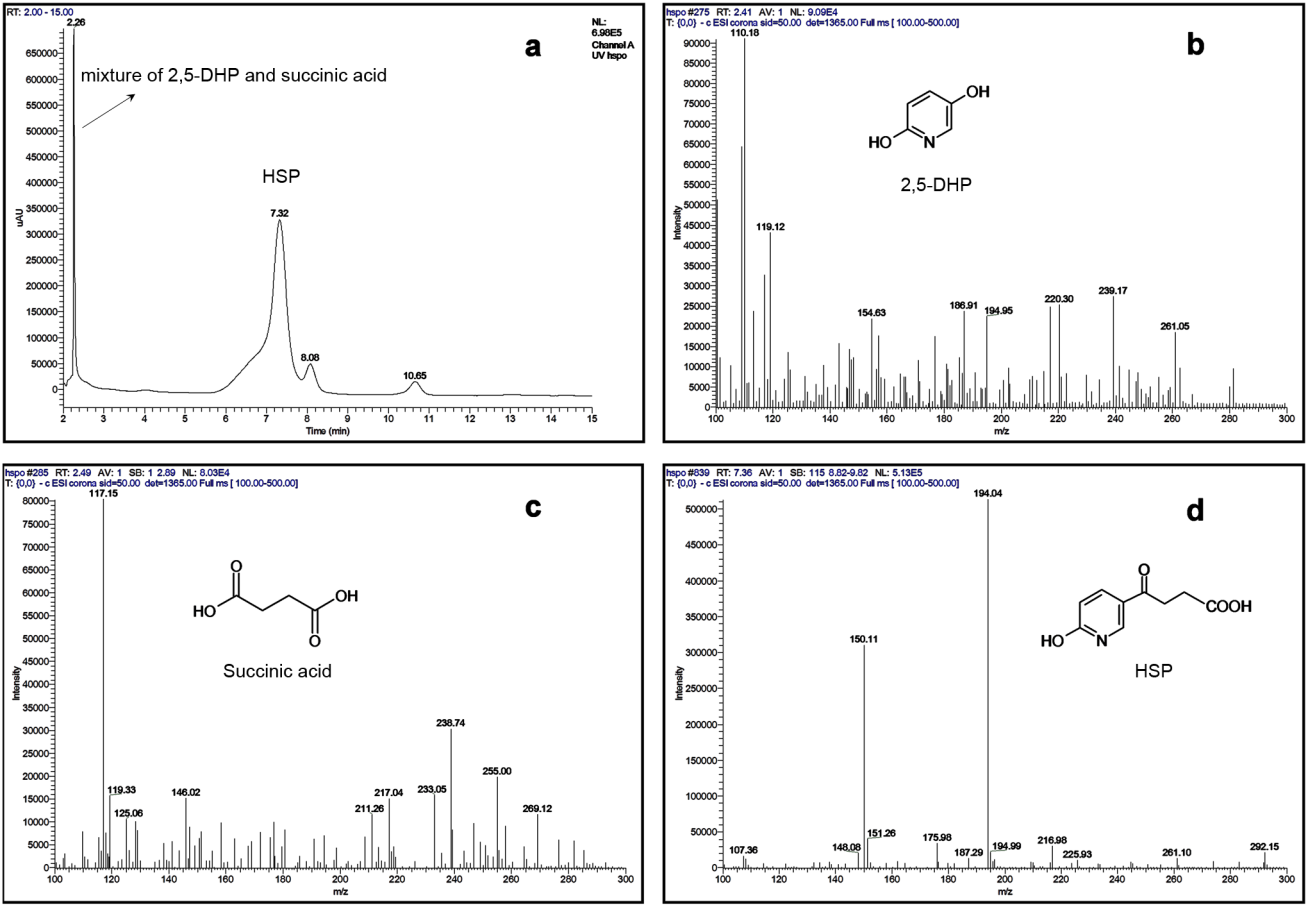
LC-MS profiles of the reaction catalyzed by purified HSP hydroxylase from *A. tumefaciens* S33. (a) HPLC profile monitored with PDA detector; (b–d) mass spectra of products 2,5-DHP (*m/z* 110.18) and succinic acid (*m/z* 117.15) and substrate HSP (*m/z* 194.04), respectively. Negatively charged ions were detected.

In order to identify the reaction catalyzed by the purified enzyme, the products of the NADH-dependent HSP oxidation reaction were determined by LC-MS (Figure 4). Three compounds were found from two peaks in the chromatography (retention time as 2.26 min and 7.32 min, respectively, Figure 4a) monitored with PDA detector by detecting negatively charged ions. The ratios of mass to charge (*m/z*) of their main fragments in the mass spectra are 110.18 (Figure 4b), 117.15 (Figure 4c) and 194.04 (Figure 4d), which fit the calculated molecular mass of 2,5-DHP (C_5_H_5_NO_2_, 111.10), succinic acid (C_4_H_6_O_4_, 118.09) and HSP (C_9_H_9_NO_4_, 195.17), respectively. Thus, the peak with retention time of 7.32 min was the substrate HSP, the peak (2.26 min) was the mixture of 2,5-DHP and succinic acid, which were difficult to separate from each other even under the best conditions we had in the liquid chromatography. However, succinic semialdehyde found in the previous report [21] was not detected even by scanning all the negatively charged ions in the spectra. One of the possible reasons may be that succinic semialdehyde produced in the reaction was oxidized into succinic acid under the reaction conditions used. In addition, the products were also analyzed with GC-MS after silylation the sample with BSTFA. The trimethylsilyl (TMS) derivatives of HSP and 2,5-DHP were able to be detected and identified, the mass of spectra of whose TMS derivative (GC-MS data not shown) were identical to the previous description [24]. Succinic acid could not be detected because of its poor separation with GC and low abundance based on the LC-MS analysis.

**Figure 5:**
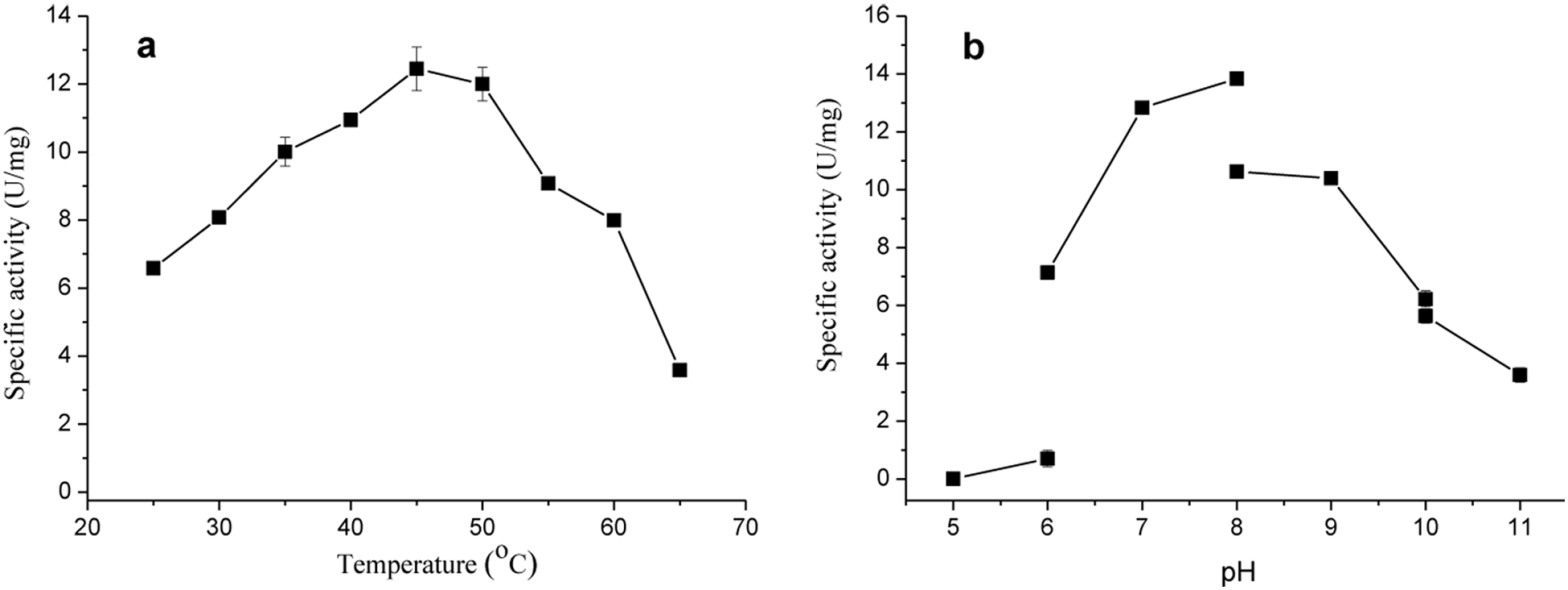
Effects of temperature and pH on the activity of HSP hydroxylase from A. *tumefaciens* S33. (a) The reactions were carried out in 50 mM Tris-HCl (pH 8.0) at the temperature indicated. (b) The reactions were performed at 30°C in different buffers: citric acid-sodium hydrogen phosphate (pH 5.0 and pH 6.0), sodium phosphate (pH 6.0, pH 7.0 and pH 8.0), Tris-HCl (pH 8.0, pH 9.0 and pH 10.0) and sodium bicarbonate-sodium hydroxide (pH 10.0 and pH 11.0). The values are means of three replicates, and the error bars indicate the standard deviations.

The purified enzyme catalyzed HSP-dependent NADH oxidation with temperature and pH optima near 45°C and pH 8.0, respectively (Figure 5). Compared with Tris-HCl buffer, the enzyme showed higher activity in phosphate buffer. Under the condition of 30°C and 50 mM phosphate buffer (pH 8.0), the enzyme presented an *V_max_* of 18.8±1.85 U/mg (the value is the mean ± SD of triplicates, the same hereinafter), an *K_m_* for HSP of 0.15±0.02 mM and an *K_m_* for NADH of 0.1±0.01 mM. NADPH could also be oxidized by HSP hydroxylase with a specific activity of 12.9±0.6 U/mg and *K_m_* of 0.35±0.04 mM. The results indicate that NADH is the preferred physiological electron donor *in vivo*.

### Transformation of HSP into 2,5-DHP

**Figure 6:**
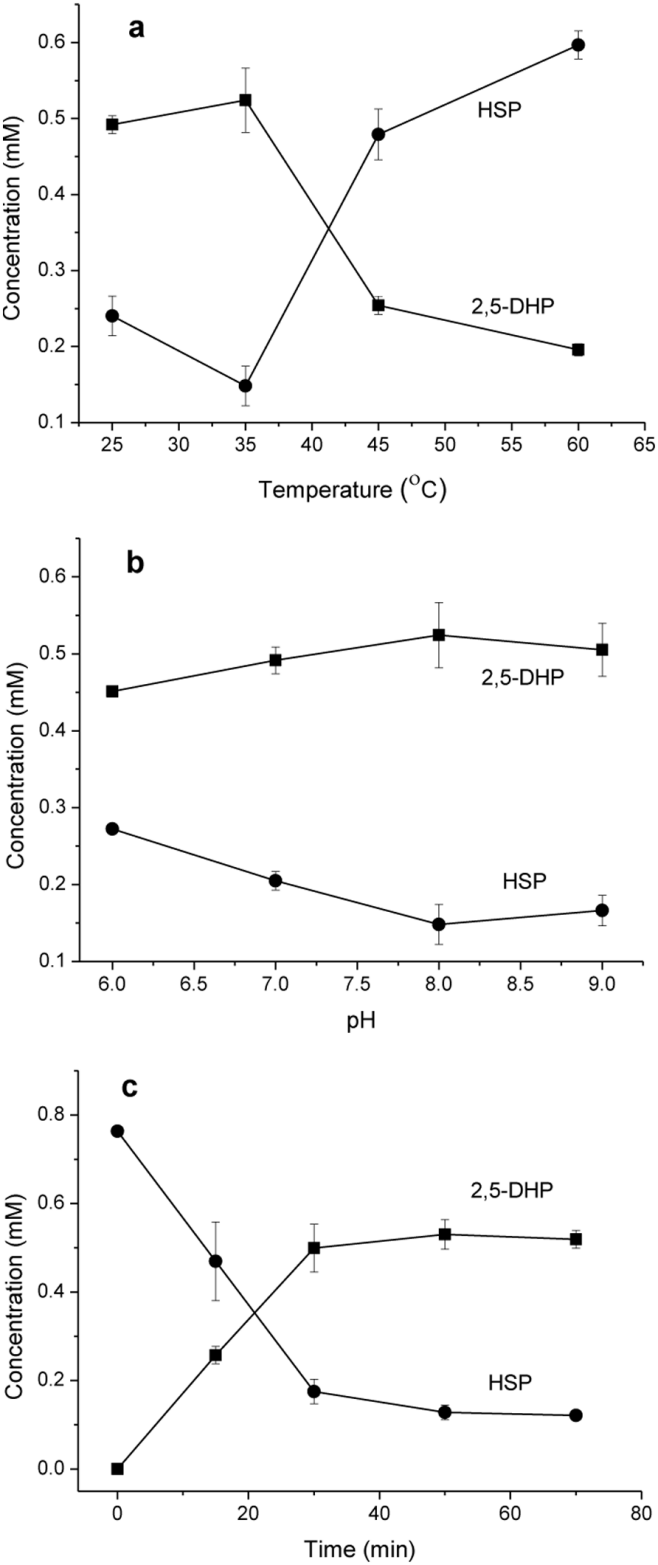
Biotransformation of HSP into 2,5-DHP by HSP hydroxylase from *A. tumefaciens* S33. Effects of temperature (a) and pH (b) on the enzymatic formation of 2,5-DHP (*squares*) from HSP (*circles*). (a) The reactions were carried out in 50 mM sodium phosphate (pH 8.0) at the temperature indicated. (b) The reactions were performed at 35°C in sodium phosphate buffer at pH indicated. (c) The reaction was performed in 50 mM sodium phosphate (pH 8.0) at 35°C. The values are means of three replicates, and the error bars indicate the standard deviations.

HSP hydroxylase from *A. tumefaciens* S33 can catalyze HSP to produce 2,5-DHP in the presence of NADH, which is a valuable precursor for synthesis of drugs and insecticides [16]. Therefore, an enzymatic route to produce 2,5-DHP from HSP was tried, which could provide an alternative for utilizing tobacco and its main alkaloid nicotine. Formate dehydrogenase has been used as an effective NADH-regenerating tool for many biocatalytic reactions [34]. In this study, it was used to supply NADH for the biotransformation of HSP into 2,5-DHP. Firstly, induced whole cells of recombinant *E. coli* BL21(DE3) harboring pETDuet-hsph and pACYCDuet-fdh was used as catalyst to convert HSP to 2,5-DHP. During the reaction, HSP was found to be consumed, however, no 2,5-DHP could be detected based on either TLC or HPLC analysis. This suggested that the product 2,5-DHP might be oxidized in the cells of *E. coli.* Further, an enzymatic transformation method was tested by using recombinant HSP hydroxylase and formate dehydrogenase. Both His-tagged enzymes were purified by His Trap HP and Source 30Q columns. The purified recombinant HSP hydroxylase showed same properties and specific activity as the wild-type enzyme purified from *A. tumefaciens* S33 (data not shown). As shown in Figures 6a and 6b, the formation of 2,5-DHP from HSP was in the condition of temperature and pH optima around 35°C and pH 8.0, respectively. Under this condition, around 0.53±0.03 mM 2,5-DHP was produced from 0.76±0.01 mM HSP after 50 min, and the molar conversion was 69.7% (Figure 6c). The amount of 2,5-DHP did not increase when incubated for longer time. On the contrary, the amount of 2,5-DHP began to decrease (data not shown) because of its oxidation in the air as indicated by the appearance of blue gray color [22], [35], which suggests that the biotransformation process should be finished as soon as possible in order to avoid product oxidation.

## Conclusion

*A. tumefaciens* S33 was previously proposed to degrade nicotine via a fused pathway of the pyridine and pyrollidine pathways. Here, HSP hydroxylase, one key enzyme involved in the pathway, was purified and characterized. The NADH-dependent flavoprotein catalyzes the oxidative decarboxylation of HSP to produce 2,5-DHP. The finding provides a new insight into the mechanism and diversity of nicotine catabolism in nature.

An enzymatic route to produce a functionalized pyridine, 2,5-DHP, from HSP was developed, which is a precursor of important chemicals and difficult to synthesize by chemical methods. This provides a new method to utilize the tobacco and its wastes.
